# Protein complex formation during denitrification by *Pseudomonas aeruginosa*

**DOI:** 10.1111/1751-7915.12851

**Published:** 2017-08-31

**Authors:** José Manuel Borrero‐de Acuña, Kenneth N. Timmis, Martina Jahn, Dieter Jahn

**Affiliations:** ^1^ Institute of Microbiology Technische Universität Braunschweig Spielmannstr. 7 Braunschweig Germany; ^2^ Braunschweig Integrated Centre of Systems Biology BRICS Technische Universität Braunschweig Rebenring 56 Braunschweig Germany

## Abstract

The most efficient means of generating cellular energy is through aerobic respiration. Under anaerobic conditions, several prokaryotes can replace oxygen by nitrate as final electron acceptor. During denitrification, nitrate is reduced via nitrite, NO and N_2_O to molecular nitrogen (N_2_) by four membrane‐localized reductases with the simultaneous formation of an ion gradient for ATP synthesis. These four multisubunit enzyme complexes are coupled in four electron transport chains to electron donating primary dehydrogenases and intermediate electron transfer proteins. Many components require membrane transport and insertion, complex assembly and cofactor incorporation. All these processes are mediated by fine‐tuned stable and transient protein–protein interactions. Recently, an interactomic approach was used to determine the exact protein–protein interactions involved in the assembly of the denitrification apparatus of *Pseudomonas aeruginosa*. Both subunits of the NO reductase NorBC, combined with the flavoprotein NosR, serve as a membrane‐localized assembly platform for the attachment of the nitrate reductase NarGHI, the periplasmic nitrite reductase NirS via its maturation factor NirF and the N_2_O reductase NosZ through NosR. A nitrate transporter (NarK2), the corresponding regulatory system NarXL, various nitrite (NirEJMNQ) and N_2_O reductase (NosFL) maturation proteins are also part of the complex. Primary dehydrogenases, ATP synthase, most enzymes of the TCA cycle, and the SEC protein export system, as well as a number of other proteins, were found to interact with the denitrification complex. Finally, a protein complex composed of the flagella protein FliC, nitrite reductase NirS and the chaperone DnaK required for flagella formation was found in the periplasm of *P. aeruginosa*. This work demonstrated that the interactomic approach allows for the identification and characterization of stable and transient protein–protein complexes and interactions involved in the assembly and function of multi‐enzyme complexes.

## Introduction

Most organisms, with the exception of a few fermentative bacteria, utilize membrane‐associated respiratory processes for cellular energy generation. During respiration, electrons are transported along a chain of redox‐active cofactors fixed to large, membrane‐associated enzyme complexes driven by the corresponding redox potentials (Borrero‐de Acuna *et al*., 2016). The linked release of energy is employed for the formation of a chemo‐osmotically active proton/sodium ion gradient, which in turn allows for ATP formation via proton/sodium ion‐driven ATP synthases (Jahn and Jahn, 2012). Multiple electron donors and acceptors can be employed by a wealth of different enzyme complexes carrying diverse redox cofactors (Marreiros *et al*., 2016). However, all these components have to be localized in the appropriate membrane, assembled with their cofactors, and possess the appropriate contacts required for productive electron transfer. It has been shown that stacking of the different complexes facilitates correct channelling and transport of electrons through the respiratory chains (Guo *et al*., 2016). The loss of energy is thereby minimized, and undesired side reactions, such as the formation of free reactive oxygen species (ROS), are reduced. There are two different hypotheses regarding the nature of the interactions that might occur between complexes I–V of the mitochondrial respiratory chain, the ‘fluid‐state’ and the ‘solid‐state’ models (Lapuente‐Brun *et al*., 2013). According to the fluid‐state model, the diverse respiratory complexes are able to diffuse without restraints along the inner mitochondrial membrane. In this case, electrons are transferred from one complex to the succeeding one when both entities randomly collide (Porras and Bai, 2015). In contrast, the solid model assumes that respiratory complexes are built in a highly organized and rigid manner (Enriquez, 2016). However, there is experimental evidence supporting co‐occurrence of elements of both models. Accordingly, it has been presumed that in a natural system, the dynamic interchange between both states is the most effective mode. This leads to the ‘plasticity’ or ‘dynamic aggregate’ model, in which the complexes can freely switch from one state to the other (Acin‐Perez and Enriquez, 2014). In this model, complexes I, III and IV strongly interact with each other creating the respirasome supercomplex, which oxidizes NADH. The respirasome in turn interacts with complex II (succinate dehydrogenase) which accepts electrons from FADH_2_. The coenzyme Q and cytochrome *c*, normally found in pools, are able to diffuse along the membrane and associate with these complexes, transferring the electrons from one complex to another (Alcazar‐Fabra *et al*., 2016). The formation of tight respirasome complexes either in eukaryotes or in prokaryotes has been demonstrated by different methods. Many higher‐ordered complexes were discovered and analysed by Blue‐native gel electrophoresis, like the respirasomes from yeast (Schagger and Pfeiffer, 2000), mouse fibroblast (Lapuente‐Brun *et al*., 2013), spinach (Krause *et al*., 2004) and potato (Bultema *et al*., 2009). Supercomplexes from bovine mitochondria were visualized with single particle cryoelectron tomography (Dudkina *et al*., 2011). Immunochemical and proteomics methods were used for the elucidation of complex composition (Borrero‐de Acuna *et al*., 2016). Similarly, several respiratory supercomplexes were analysed in bacteria like the sulfide oxidase–oxygen reductase from *Aquifex aeolicus* (Prunetti *et al*., 2010) and the respirasome from *Paracoccus denitrificans* (Stroh *et al*., 2004). The dynamics of oxidative phosphorylation complexes in *Eschericha coli* was recently discussed (Magalon *et al*., 2012; Magalon and Alberge, 2016). However, knowledge of the assembly, composition, function and degradation of all these complexes is limited.

## The denitrification machinery of *Pseudomonas aeruginosa*

The ubiquitously found, metabolically highly versatile bacterium *Pseudomonas aeruginosa* is proliferates in diverse environments, such as soil, water and even on the surfaces of hospital equipment (Talwalkar and Murray, 2016). It is also an important opportunistic pathogen and one of the most predominant pathogens causing acute and chronic lung infections in immunocompromised hosts (Oliver *et al*., 2000; Driscoll *et al*., 2007; Auerbach *et al*., 2015). *P*. *aeruginosa* is a facultative anaerobe able to respire nitrate or nitrite and to ferment pyruvate and arginine when oxygen becomes exhausted (Vander Wauven *et al*., 1984; Eschbach *et al*., 2004). The capability of thriving at low oxygen partial pressure facilitates the invasion of the mucus of cystic fibrosis patients (Alvarez‐Ortega and Harwood, 2007). Worldwide, there are over 70.000 cases of *P*. *aeruginosa* infections of patients with this ion‐channel‐defect genetic disease (Aloush *et al*., 2006). *P*. *aeruginosa* replaces molecular oxygen as terminal electron acceptor for the respiratory chain with different N‐oxides during anaerobiosis. An electron transport with the terminal enzyme nitrate reductase converts nitrate into nitrite, generates a proton/sodium ion gradient and synthesizes ATP. Similarly, nitrite is reduced to NO, N_2_O and further to molecular nitrogen by 3 further respiratory chains with the terminal enzymes nitrite, NO and N_2_O reductase (Zumft, 1997). Two of these reductases (nitrite reductase and N_2_O reductase) are localized in the periplasm, and the other two (nitrate reductase and NO reductase) reside in the inner membrane (Schobert and Jahn, 2010). Three regulatory systems control the onset of denitrification (Schreiber *et al*., 2007; Trunk *et al*., 2010). The Fnr/Crp‐type regulator Anr (PA1544) senses the absence of oxygen, while the two‐component regulatory system NarXL (PA3878; PA3879) monitors nitrate, and a second Fnr/Crp‐type regulator Dnr (PA0527) detects the formation of NO (Pessi and Haas, 2000; Rinaldo *et al*., 2005). In a regulatory cascade reaction, Anr, Dnr and NarXL gradually induce the denitrification operon (Van Alst *et al*., 2007). Many of the denitrification proteins produced are exported via the Sec protein secretion system into the periplasm (Denks *et al*., 2014). The incorporation of essential cofactors (Fe‐S‐clusters, molybdenum cofactor, haem, metals) requires their synthesis, transport and insertion by specialized proteins (Blasco *et al*., 2001; Magalon and Mendel, 2015; Dailey *et al*., 2017). To elucidate the protein–protein interaction network involved in the formation of the denitrification apparatus, a proteomics‐based interactomic approach was combined with electron microscopy (Borrero‐de Acuna *et al*., 2016).

## The proteomics‐based interactomic approach

First, bait proteins were selected on the basis of results from physiological experiments. For example, mutants in the genes for NO reductase subunits B (PA0524) and C (PA0523); (NorBC) and the flavoprotein NosR (PA3391) revealed a nitrate reductase‐deficient growth phenotype (Borrero‐de Acuna *et al*., 2016). However, an intact nitrate reductase was detected in these mutants using appropriate antibodies. Thus, NorBC and NosR were presumed to functionally interact with nitrate reductase to form a higher‐ordered protein complex anchored to the inner membrane (Vaccaro *et al*., 2015; Cutruzzola and Frankenberg‐Dinkel, 2016; Zhang *et al*., 2017). NorBC and NosR were therefore selected as bait proteins for the interactomic experiments. Another interesting physiological observation was the failure of *P*. *aeruginosa* nitrite reductase (NirS) gene mutants to build an intact flagellum resulting in the loss of swimming motility (Borrero‐de Acuna *et al*., 2015). The periplasmic NirS was therefore selected as a bait protein to identify its interaction partners, using affinity chromatography purification coupled with mass spectrometry (Borrero‐de Acuna *et al*., 2015).

**Figure 1:**
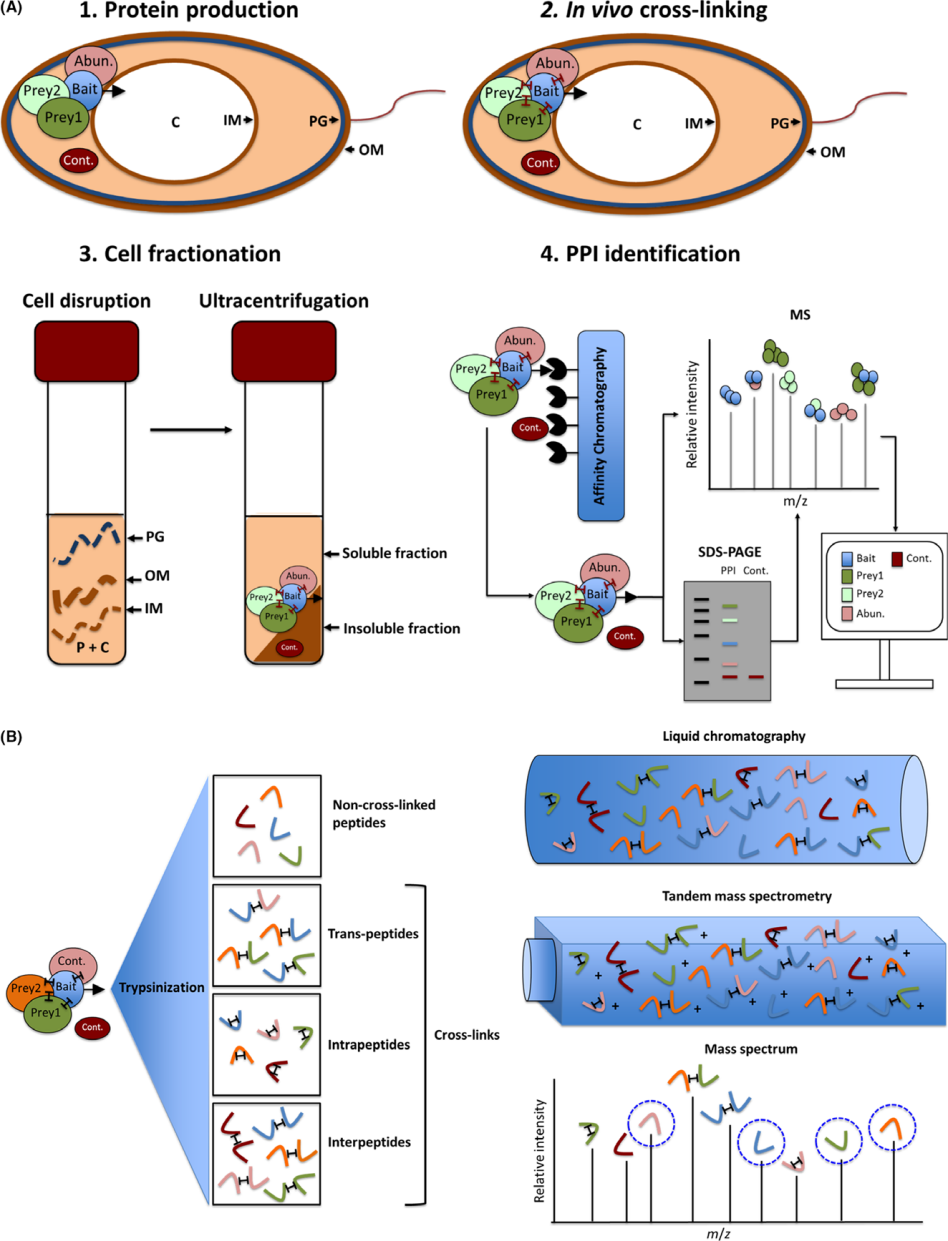
General interactomic workflow. The illustration depicts step by step the protein–protein interaction elucidation pathway via affinity chromatography copurification coupled with mass spectrometry. A. Shows: (1) Induction of the bacterial host for bait–protein production by the use of anaerobic growth condition in the presence of nitrate; (2) the *in vivo* cross‐linking by the addition of diffusible cross‐linkers; (3) the cell fractionation and separation of in‐ and soluble fraction by centrifugation; and in (4) the affinity tag‐based purification of the formed and cross‐linked protein complexes, their visualization on an SDS–PAGE gel, the mass spectrometry analyses of corresponding peptides and the computational identification of bait interaction partner candidates. B. shows the downstream processing of cross‐linked peptides. There are four possible varieties of resulting peptides after trypsin digestion: non‐cross‐linked peptides, interpeptides (cross‐links between diverse peptides of the same protein), intrapeptides (cross‐links within the same peptide of a certain protein) and trans‐peptides (cross‐links between peptides of different proteins). The LC‐MS/MS analysis is depicted in detail. Abun., Abundant proteins that unspecifically attach to the bait–prey complex (identified by the analysis of their protein abundance prior to affinity purification enrichment. Thus, proteins enriched after purification are considered as potential interaction partners); PPI, protein–protein interactions; Cont., contaminant with affinity to the column material (detected with a control strain lacking the expression plasmid); MS, mass spectrometry; PG, peptidoglycan; C, cytoplasm; IM, inner membrane; OM, outer membrane.

The workflow of affinity copurification of prey proteins with the corresponding bait proteins, coupled to prey protein identification by LC‐MS/MS, is shown in Fig. 1A. Denitrifying conditions were achieved by anaerobically incubating nitrate‐supplemented cultures until the late exponential phase (Galimand *et al*., 1991). At this point, protein cross‐linking through addition of formaldehyde was carried out to stabilize scarce and transient protein–protein interactions within the protein complexes. The new peptides, i.e. peptides not found in the native proteins, created by cross‐linking and released by trypsin treatment, are shown in Fig. 1B. LC‐MS/MS analyses readily identified non‐cross‐linked peptides, whereas inter‐ and trans‐peptides posed problems for identification because the corresponding m/z ratios were shifted (Fig. 1B). This observation was used for the identification of the interacting domains of two proteins (NirS and FliC) as outlined below. Due to the chemical nature of this cross‐linker, its penetration through cellular membranes is rather fast (20 min). Furthermore, formaldehyde cross‐linking preserves the native structures of the proteins in the formed complexes. Cross‐linking was quenched by adding an amino acid such as glycine. Formed formaldehyde‐based cross‐links were disrupted again by subjecting the samples to high temperatures (95°C) for short periods of about 20 min (Sutherland *et al*., 2008), thereby allowing recovery of analysable peptides for mass spectrometry analysis. However, this method does not distinguish between directly bait‐bound proteins and proteins indirectly bound via other bait‐bound polypeptides.

Due to the different cellular locations of our bait proteins, NirS in the periplasm and NorBC/NosR in the membrane, different preparation procedures for the isolation of the cross‐linked complexes and the corresponding background control samples were established. Periplasmic proteins were released by an osmotic shock treatment by addition of 300 mM sucrose, avoiding complete cell disruption and minimizing release of cytoplasmic proteins, followed by ultracentrifugation (100 000 × *g*) (Nicke *et al*., 2013). Membrane proteins were obtained by French Press disruption, followed by ultracentrifugation to separate soluble from insoluble proteins. Membrane proteins were subsequently solubilized by addition of Triton X‐100 for further purification. Afterwards, all isolated protein fractions were purified by affinity chromatography with stringent washing to eliminate contaminants (see Fig. 1) (Makowski *et al*., 2016). Contaminants consist of proteins interacting with the column material. Periplasmic and membrane protein fractions prepared in parallel from the parental strain without bait served as background controls.

Affinity‐purified protein complexes were subject to quantitative LC/MS‐MS‐based proteomics. If a prey was measured in larger amounts in the protein complex in comparison with the natural appearance in the proteomic analysis of the disrupted *P*. *aeruginosa,* prior to affinity chromatography purification (control), its interaction with the bait was considered to be specific. For this purpose, the abundance of a specific protein was measured by elucidating the average area of its three most prominent mass spectrometry peaks in the sample before and after affinity purification. A semi‐quantitative value was given to each polypeptide according to this average area that directly reflects protein abundance. The abundance of each protein prior and subsequent to affinity purification was then compared (Ong and Mann, 2005; Kaake *et al*., 2010; Howden *et al*., 2013). The software Proteome Discoverer was employed for m/z analysis and identification of interaction partner candidates (Al Shweiki *et al*., 2017).

Tandem affinity purification has proven useful for precise protein interaction partner determination, avoiding the presence of contaminants to a high extent (Burckstummer *et al*., 2006). Here, the use of just one tag combined with the application of stringent washing steps led to highly accurate results. In our approach, different types of affinity tags, i.e. His6‐tag as well as the Strep‐tag II, were employed. The gene encoding the periplasmic protein NirS was genetically fused to Strep‐tagII, whereas membrane protein encoding genes *norC*,* norB*,* nosR* were fused to the His6‐tag. Expression of all of these genes was driven by the cognate native promoters and thereby controlled by the intrinsic transcriptional mechanisms of *P*. *aeruginosa*. The identification of potential N‐terminal signal peptides of the bait proteins is crucial to properly locate the selected tag (Dalbey and Kuhn, 2012). C‐terminal tags should not interfere with protein translocation. The tagged bait proteins were produced in mutant backgrounds eliminating the non‐tagged counterpart of the protein of interest (i.e. *norC*‐His6‐tag in a transposon *norC* mutant).

## The antibody‐based interactomic approach

Identified protein–protein interactions were confirmed by a second, technically independent, approach. In this case, antibodies against the potential interaction partners were obtained and used for *in vivo* colocalization experiments by electron microscopy. Polyclonal antibodies against the different baits used (NorB, NorC, NosR, NirS) and preys detected (NarH, DnaK, FliC) were raised. DnaK and NosZ antibodies were synthesized using the pET14b plasmid and *E. coli* BL21. Specific antibodies against NarH, NosR, NirS, NorC and FliC were produced by immunization using peptides representing specific soluble domains of the proteins of interest, namely peptides spanning amino acid residues 50 to 61, 102 to 116, 118 to 132 and 106 to 130, for NorC; 190 to 203 and 404 to 418 for NosR; 1 to 276, 277 to 392, and 418 to 513, for NarH; 379 to 392, 526 to 540, and 541 to 555, for NirS; and the first 174 amino acids, for FliC. In the case of membrane proteins NarH, NosR and NorC, the chosen peptides were hydrophilic representing loops of the protein protruding into the cytoplasm or periplasm. Based on the known crystal structures of the soluble proteins NirS and FliC, peptides known to be exposed at the protein surface were preferred (Cutruzzola *et al*., 2001; Sun *et al*., 2002; Song and Yoon, 2014); (PDB: 1GJQ and PDB: 4NX9).

Obtained antibodies were conjugated to immunogold particles of different sizes (10 and 15 nm) and used to visualize by transmission electron microscopy potential colocalization of protein targets in individual cells. Both the cellular locations of individual target proteins, and their interaction partners, were thereby visualized at the single cell level (Masoumi *et al*., 2008; Borrero‐de Acuna *et al*., 2016).

## NorCB and NosR are the membrane‐based assembly platform for the denitrification Supercomplex

**Figure 2:**
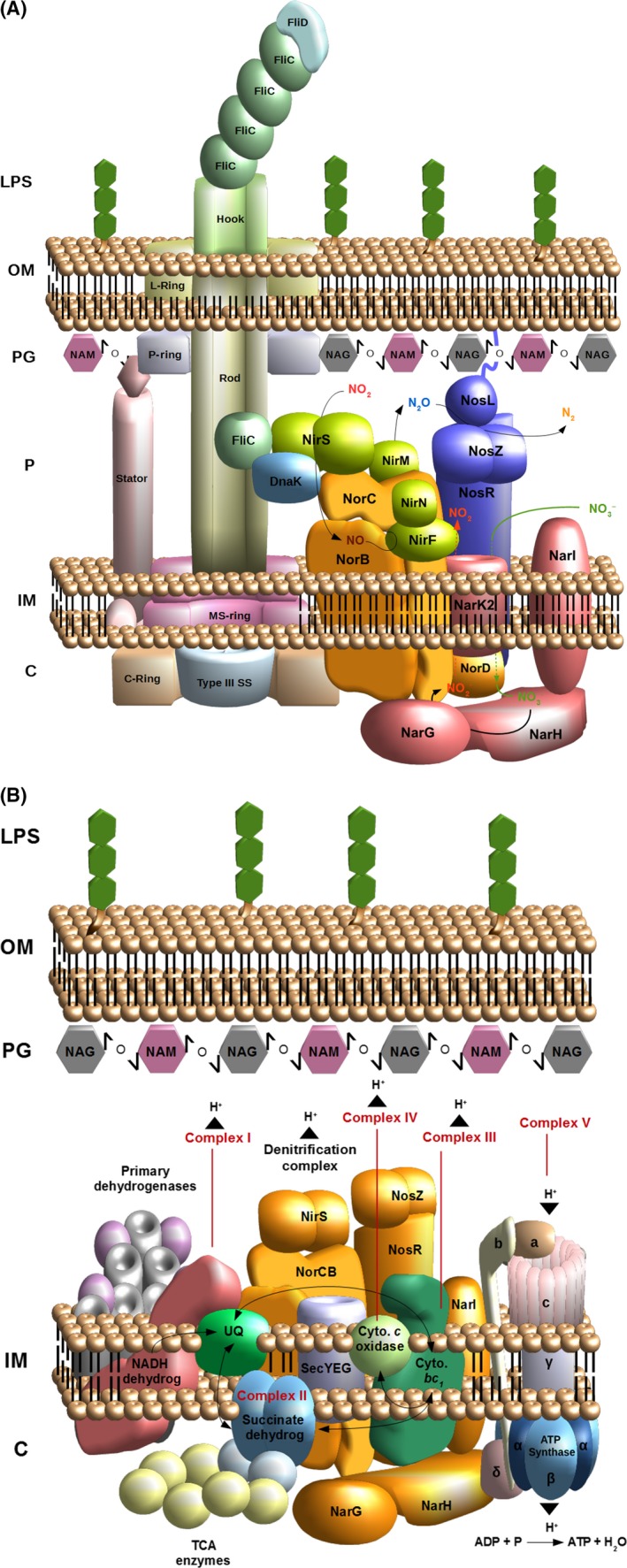
Illustration of the detected denitrification supercomplexes. A. The protein interaction partners for NirS, NosR, NorC and NorB that are related to denitrification or flagellum assembly (FliC and DnaK) are shown. The four reduction steps ${\text{NO}}_{3}^{-}$ → ${\text{NO}}_{2}^{-}$; ${\text{NO}}_{2}^{-}$ → NO; NO → N_2_O and N_2_O → N_2_ are represented. The flagellum structure is drafted based on previous works on *Salmonella enterica* and *E. coli*. B. The respirasome of *Pseudomonas aeruginosa* encompassing, among others, primary dehydrogenases, respiratory chain complexes I‐V, ubiquinol, cytochrome *c* and the SecAYEG translocon. The electron flow is specified with black arrows. The proton translocation associated with ATP generation is also depicted. NAM,* N*‐acetylglucosamine; NAG,* N*‐acetylmuramic acid; LPS, lipopolysaccharide; OM, outer membrane; PG, peptidoglycan; P, periplasm; IM, inner membrane; C, cytoplasm; type III SS, type III secretion system; TCA enzymes, enzymes involved in the tricarboxylic acid (TCA) cycle.

*Pseudomonas aeruginosa* was grown under anaerobic, denitrifying conditions. Protein interaction partners of NorBC and NosR were determined using the outlined comparative proteomics approach. Selected protein–protein interactions were visualized using the antibody‐based electron microscopy approach as described above. The robustness of our approach was demonstrated by the strong enrichment of NorB by the tagged NorC bait and *vice versa*. NosR was encountered as strong interaction partner for both proteins (Fig 2A).

The nitrate reductase NarGH (PA3875; PA3874) subunits firmly interacted with NorCB, whereas membrane‐spanning NarI (PA3872) was not found to be involved in the interaction. The nitrate/nitrite antiporter NarK2 (PA3876), responsible for nitrate import into the cytoplasm, and the parallel export of formed nitrite, interacted with NorC. This transporter was found to be essential for denitrification by *P. aeruginosa* (Sharma *et al*., 2006). A second known nitrate transporter, NarK1 (PA3877), was not found in the protein complex. The next step of denitrification is the reduction of nitrite to NO by the nitrite reductase NirS. NirF (PA0516), a protein involved in nitrite reductase (NirS) maturation via the incorporation of haem *d*_1_ into the enzyme (Bali *et al*., 2010), interacted strongly with NorCB. Furthermore, the protein NirN (PA0509) was also part of NorBC interactome (Adamczack *et al*., 2014). Finally, NirS was found in complex with NorB. These findings are consistent with the previous observation that NirS forms a maturation triad, a stable triple complex, with NirF and NirN (Fig. 2A) (Nicke *et al*., 2013). Strong interactions between NirM (PA0518), a *c*‐type cytochrome donating electrons to NirS, and NorC were also observed (Hasegawa *et al*., 2001). Furthermore, azurin (PA4292), also known as cytochrome *c*_551_, which transfers electrons from the cytochrome *bc*_1_ complex to terminal reductases of denitrification (Williams *et al*., 2007; Santini *et al*., 2014), was incorporated into this protein complex by its contact to NorC. Finally, NorC exhibited significant affinity to NirQ (PA0520), a regulatory protein required for formation of the denitrification machinery (Hayashi *et al*., 1998).

NosR is an iron–sulfur protein harbouring a flavin cofactor facing the periplasm and redox centres located on the cytoplasmic side of the inner membrane (Wunsch and Zumft, 2005) and, as indicated above, is tightly bound to the respirasome via NorC (Fig. 2A). NosR was, itself, found to interact with NarG, NarH, NirE (PA0510), NirQ, NirS and NirM. Importantly, the N_2_O reductase NosZ (PA3392) exhibited a strong interaction with NosR. The thus far not well understood NosR function seems to be linked to NosZ maturation (Cuypers *et al*., 1992; Arai *et al*., 2003; Wunsch *et al*., 2003). NosR also associates with NosL (PA3396), a lipoprotein attached to the outer membrane described as a copper‐binding protein. This protein is probably responsible for the insertion and coordination of the multicopper centre within NosZ (McGuirl *et al*., 2001; Taubner *et al*., 2004). Therefore, the N_2_O reduction machinery appears to interact with the rest of the denitrification complex principally via the NosR membrane protein and not via NorCB (Fig. 2A).

Denitrification protein colocalization studies were carried out with pairs of antibodies, raised against NarH, NirS, NorC, NosZ an NosR and subsequently conjugated to 15‐ or 10‐nm gold particles (Rohde *et al*., 2003) that were applied to ultrathin sections of *P. aeruginosa* and visualized by electron microscopy. When the observed distance between two different sized gold particles was < 25 nm, it was assumed that the two corresponding proteins had been colocalized, and hence interacted with one another (Elamin *et al*., 2011). The results of these colocalization experiments were highly consistent with the conclusions of the proteomics experiments. An important control for the colocalization approach was that when two proteins were known to be separated by the inner membrane, as is the case for cytoplasmic NarH and periplasmic NirS or NosZ, colocalization was not observed.

These observations explain results of our previous physiological experiments, in which *norB* mutants exhibit impaired nitrate and nitrite reduction *in vitro* and *in vivo*, namely that the absence of NorBC results in destabilization of the respirasome in such a way that NarGHI and NirS are unable to conduct their enzymatic functions. While NarH can still be detected in corresponding mutant bacteria, NirS cannot and hence must be prone to proteolytic degradation.

## The denitrification supercomplex as platform for the attachment of the corresponding electron transport chains

Several primary dehydrogenases were found tightly bound to the NorCB‐NosR platform. The most significant interaction partners detected were NADH dehydrogenase Nuo (Platt *et al*., 2008) (PA2638 to PA2644), proline dehydrogenase PutA (PA0782) (Nakada *et al*., 2002), l‐lactate dehydrogenase (PA4771) (Eschbach *et al*., 2004) and the D‐amino acid dehydratase (PA3357) (Ikeno *et al*., 2006). Diverse subunits of the F_o_F_1_ ATP synthase (PA5553 to 5560) (Cook *et al*., 2014) were also linked to the respiration complex. Surprisingly, several enzymes mediating the TCA cycle, like malate: quinone oxidoreductase (PA3452), succinate dehydrogenase (PA1582 to PA1584), isocitrate dehydrogenase (PA2624), citrate synthase (PA1580), succinyl‐coenzyme A (succinyl‐CoA) synthetase (PA1588) and 2‐oxoglutarate dehydrogenase (PA1585), were also found to be part of the supercomplex, as were enzymes related to the pyruvate metabolism, such as various subunits of pyruvate dehydrogenase (PA5015), pyruvate kinase (PA4329), phosphopyruvate hydratase (PA3035), acetyl‐CoA carboxylase (PA3112, PA3639 and PA4848) and phosphoenolpyruvate synthase (PA1770) (Ornston, 1971; Meylan *et al*., 2017) (Fig. 2B).

## The denitrification supercomplex as a platform for corresponding transport and maturation factors

The respirasome included several enzymes involved in haem biosynthesis, like the haem biosynthesis‐associated protein (PA5257), the potential enzyme of haem biosynthesis HemX (PA5258), coproporphyrinogen III dehydrogenase HemN (PA1546), coproporphyrinogen III oxidase HemF (PA0024) and porphobilinogen synthase HemB (PA5243) (Dailey *et al*., 2017). Furthermore, the haem d_1_ biosynthesis proteins NirF (PA0516), NirJ (PA0511), NirL (PA0514) and NirE (PA0510) were found attached (Layer *et al*., 2010). Haem d_1_ is a cofactor of nitrite reductase NirS. The electron donor systems for NirS, the *c‐*type cytochromes NirM (PA0518) and NirN (PA0509) were also associated with the complex, as were cytochrome *c*_1_ (PA4429), cytochrome *c*_5_ CycB (PA5300), cytochrome *c*_4_ precursor Cc4 (PA5490), cytochrome *c* oxidase of the *cbb3*‐type (PA1552‐4), and the cytochrome *c*‐type biogenesis proteins CcmE (PA1479) and CycH (PA1483) (Williams *et al*., 2007). Proteins involved in the biogenesis of iron–sulfur clusters, like NfuA (PA1847) (Roche *et al*., 2013), of the molybdenum cofactor, like MoaB1 (PA3915) (Andreae *et al*., 2014; Kasaragod and Schindelin, 2016; Fernandez‐Barat *et al*., 2017), and of ubiquinone, like UbiE (PA5063) (Jacewicz *et al*., 2013; Aussel *et al*., 2014), were also found in the complex. Finally, multiple proteins constituting the SecAYEG translocon, like YajC (PA3822), SecA (PA4403), SecD (PA3821), SecF (PA3820) and YidC (PA5568), were identified as interaction partners (Dalbey and Kuhn, 2012; Kudva *et al*., 2013). This is consistent with the need to secrete most of the proteins involved in denitrification to the periplasm.

## The periplasmic NirS‐DnaK‐FliC complex is essential for flagellum formation

**Figure 3:**
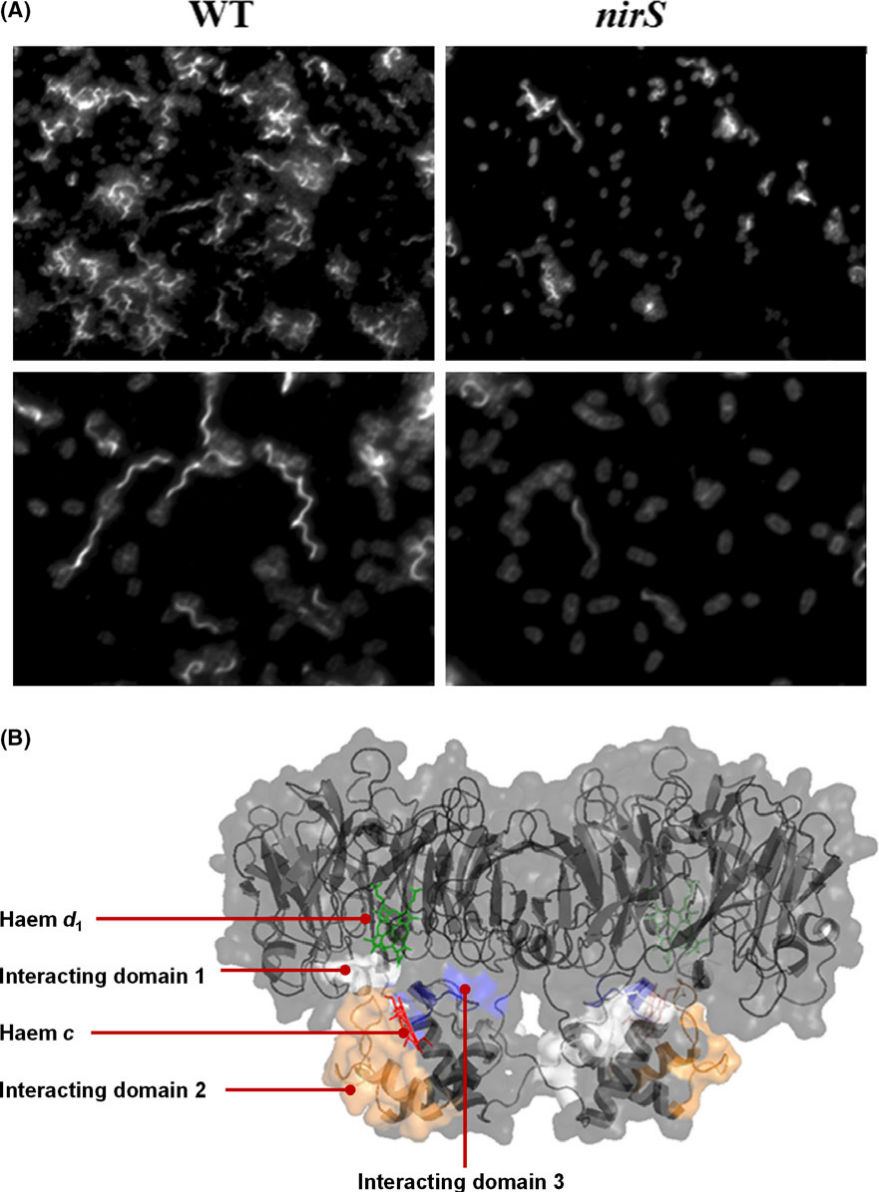
Visualization of impaired flagellar formation in the *Pseudomonas aeruginosa nirS* mutant and determination of the interacting domains of the NirS and FliC proteins by LC‐MS/MS. A. The flagellar formation in the wild type and *nirS* strains (grown under anaerobic conditions and 20 mM arginine) is shown by fluorescence microscopy. Antibodies against DnaK and FliC were raised and employed for detection. Goat anti‐rabbit Alexa 488 (for FliC) or goat anti‐rabbit Alexa 568 (for DnaK) were used as fluorescently labelled secondary antibodies. DAPI dye was utilized for DNA staining. B. The interacting domains between NirS and FliC elucidated by mass spectrometry by determination of the cross‐linked peptides are shown for NirS: AAEQYQGAASAVDPTHVVR (white), CAGCHGVLRK (blue) and GQQYLEALITYGTPLGMPNWGSSGELSK (orange). The haem *d*_1_ (green) and haem *c* (red) are also highlighted in the NirS structure.

During elucidation of interaction partners for NirS, a triad constituted of NirS‐DnaK‐FliC was found in the periplasm of *P. aeruginosa* by the proteomics approach and subsequently confirmed by colocalization analyses (Borrero‐de Acuna *et al*., 2015), suggesting a role of the respirasome in motility. Consistent with this suggestion, the *nirS* transposon mutant exhibited swimming impairment in swimming motility assays, and electron microscopic examination of the mutant revealed defective flagellum formation (Fig. 3A). This finding raised the question of whether the role of NirS in motility is structural or enzymatic, i.e. whether or not the nitrite reductase activity of NirS is essential for flagellum formation and motility. We therefore examined the swimming ability of a *nirF* mutant, because NirF is required for NirS maturation, and a *nirF* mutant produces an intact NirS protein lacking nitrite reductase activity (Adamczack *et al*., 2014). This experiment revealed that the *nirF* mutant exhibited a normal flagellum and mobility, indicating a structural role of NirS in flagellum formation (Borrero‐de Acuna *et al*., 2015). As crystal structures of FliC and NirF are available, their contact surfaces in the triad could be identified by means of the proteomics approach. Detection of cross‐linked FliC‐NirS peptides, and comparison with their un‐cross‐linked counterparts revealed that NirS and FliC interacted via surfaces involving the AAEQYQGAASAVDPTHVVR, CAGCHGVLRK and GQQYLEALITYGTPLGMPNWGSSGELSK peptides of NirS (Fig. 3B) and the NQVLQQAGT, AILAQANQLPQAVLSLLR and LGITASINDK peptides of FliC (Fig. 3B). Although the presence of the DnaK in the triad and in the periplasm was unambiguously documented by electron microscopy (Borrero‐de Acuna *et al*., 2015), its role in the triad remains to be determined.

## Conclusion and outlook

A dynamic supercomplex for anaerobic denitrification is formed at a membrane‐localized platform via transient and stable protein–protein interactions ensuring efficient electron transfer for ATP generation. Essential complex maturation and control proteins are intrinsic to the complex. Moreover, enzymes of this supercomplex also serve for unexpected structural purposes during flagellum assembly. It seems that we presently only have a glimpse of a novel protein–protein interaction world coordinating the structural assembly and function of protein complexes serving central cellular processes at the cytoplasmic membrane. The question of the mechanisms of complex disassembly and component breakdown or recycling during adaptation to new environmental conditions, as occurring during oxygen respiration, will be a research focus of the future. How are membrane‐associated transport and signal perception processes included in the observed protein complex dynamics? How is this membrane activity coordinated with cytoplasmic activities? Clearly, we have to think complex.

## Conflict of Interest

None declared.
