# Corrigendum

**DOI:** 10.1111/1751-7915.13048

**Published:** 2018-02-14

Borrero‐de Acuña, J.M., Timmis, K.N., Jahn, M., and Jahn, D. (2017) Protein complex formation during denitrification by Pseudomonas aeruginosa. *Microb Biotechnol* 10: 1523–1534.

In the original publication (*Microbial Biotechnology*, 2017; 10: 1523–1534), the sources of Figure 3 were not properly acknowledged. The Acknowledgements section should read:

‘This work was supported by ERC grant IPBSL, awarded to K.N.T. and Ricardo Amils and the funding by the Deutsche Forschungsgemeinschaft (Forschergruppe PROTRAIN) granted to M.J. and D.J. We are grateful to Dr. Manfred Rohde and Dr. Gabriella Molinari at the Central Facility for Microscopy, Helmholtz Centre for Infection Research, Braunschweig for providing the microscopic images.’

The authors sincerely apologize for the confusion caused by this error.

